# Building community networks and engagement for effective TB case management

**DOI:** 10.3389/fpubh.2025.1576875

**Published:** 2025-05-29

**Authors:** Augustine Kumah

**Affiliations:** ^1^The Bank Hospital, Accra, Ghana; ^2^Research on Interventions for Global Health Transformation – RIGHT Institute, Accra, Ghana

**Keywords:** tuberculosis case management, community networks, community engagement, TB control, public health

## Abstract

Tuberculosis (TB) remains a significant global public health challenge, particularly in low- and middle-income countries. Effective TB case management requires comprehensive strategies that extend beyond clinical treatment and involve community engagement and networks. This narrative review explores the role of community networks and engagement in enhancing TB case management, focusing on how communities contribute to improved detection, treatment adherence, and long-term management. Drawing on a wide range of studies, the review highlights the importance of participatory approaches, the use of community health workers (CHWs), and multi-sectoral collaboration in TB care. It emphasizes the role of culturally tailored interventions and the need for greater investment in building sustainable community networks. The discussion also explores the challenges and barriers to effective community engagement, such as stigma, lack of resources, and structural inequalities. The review concludes by proposing recommendations for future strategies to strengthen community networks and engagement, ensuring more comprehensive and effective TB case management.

## Introduction

Tuberculosis (TB) is a preventable and usually curable disease (1, 2). Yet in 2023, TB probably returned to being the world’s leading cause of death from a single infectious agent, following 3 years in which it was replaced by coronavirus disease (COVID-19) and caused almost twice as many deaths as HIV/AIDS (2). More than 10 million people continue to fall ill with TB every year, and the number has been rising since 2021 (2).

TB is caused by the bacillus *Mycobacterium tuberculosis*, which is spread when people who are sick with TB expel bacteria into the air (e.g., by coughing) (1). Approximately a quarter of the global population is estimated to have been infected with TB (2). Following infection, the risk of developing TB disease is highest in the first 2 years (approximately 5%), after which it is much lower (1–3). Of the total number of people who develop TB disease each year, approximately 90% are adults, with more cases among men than women (1, 2). The disease typically affects the lungs (pulmonary TB) but can also affect other sites (1, 2, 4).

While TB is curable and preventable, the disease persists due to several factors, including socio-economic barriers, stigma, and the difficulties associated with prolonged treatment regimens. Despite global efforts, TB control remains a challenge, particularly in low-resource settings where healthcare infrastructure is weak, and patients often experience barriers to accessing timely diagnosis and treatment (5, 6). Effective TB case management requires robust medical interventions and a strong focus on community engagement and building networks that support case identification, treatment adherence, and follow-up care (7, 8).

Community engagement has been increasingly recognized as vital in the global fight against TB. Building on social capital, community networks can help identify TB cases earlier, support patients throughout their treatment journey, and reduce stigma, which remains a significant barrier to care (9–11). The engagement of community health workers (CHWs), volunteers, local leaders, and non-governmental organizations (NGOs) can bridge gaps between formal healthcare systems and the communities they serve, enhancing TB case management through locally adapted solutions. Furthermore, involving communities in designing and implementing TB control programs promotes ownership and sustainability (9, 10, 12, 13).

This study explores the global incidence of TB; the global progress made in TB prevention and treatment and discusses how community networks and engagement contribute to effective TB case management; the role of community networks in TB case detection, how community engagement can support treatment adherence; how community interventions could reduce TB-related stigma and multi-sectoral collaboration; and sustainability for effective TB prevention and management. This review provides insight into how these strategies can strengthen the role of communities in TB management, contributing to improved health outcomes.

## Methods

**Figure 1:**
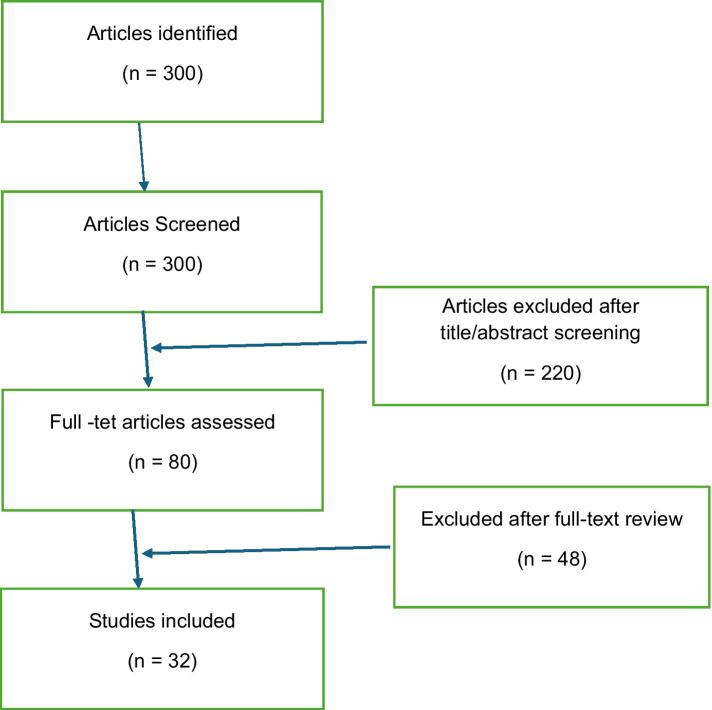
PRISMA diagram: selection of articles.

A literature search was conducted on community engagement in TB case management. The search was conducted across multiple academic databases, including PubMed, Scopus, and Google Scholar, covering 2000 to 2023. The key search terms used included “community engagement in tuberculosis,” “community health workers and TB,” “TB case management,” “community networks in healthcare,” and “TB stigma and community.” Articles included in the review were peer-reviewed journal articles, systematic reviews, and reports from reputable international organizations such as the WHO and the Stop TB Partnership. The inclusion criteria for the review were studies that discussed the role of communities in TB case management, provided insights into interventions involving community health workers, and examined the impact of community-based approaches on TB detection, treatment adherence, and outcomes. The initial search yielded over 300 articles. After screening titles and abstracts for relevance, 80 full-text articles were reviewed, and 32 studies were included in the final analysis. The selected studies were analyzed thematically, focusing on key areas such as community participation in TB care, the use of community health workers, stigma reduction, and the role of multi-sectoral collaboration. The review also considered studies that examined barriers to community engagement and strategies to overcome these challenges (Figure 1).

## Results

### Global incidence of TB: number of people developing TB

A global total of 8.2 million people were reported as newly diagnosed with TB in 2023, up from 7.5 million in 2022 and 7.1 million in 2019 and far above the levels of 5.8 million in 2020 and 6.4 million in 2021. Those newly diagnosed in 2022 and 2023 probably included a sizeable backlog of people who developed TB in previous years but whose diagnosis and treatment were delayed by COVID-related disruptions. The global gap between the estimated number of people developing TB (incident cases) and the reported number of people newly diagnosed with TB (notified cases) narrowed to a best estimate of 2.7 million in 2023, down from about 4 million in 2020 and 2021 and below the pre-pandemic level of 3.2 million in 2019.

The majority of the global increase in cases between 2022 and 2023 reflects population growth. The TB incidence rate (new cases per 100,000 population) in 2023 was 134 (95% UI: 125–145), a very small (0.2%) increase compared with 2022. The majority of the people who develop TB disease each year are in 30 high-burden countries, which accounted for 87% of the global total in 2023. Five countries accounted for 56% of the worldwide total: India (26%), Indonesia (10%), China (6.8%), the Philippines (6.8%), and Pakistan (6.3%). In 2023, 55% of people who developed TB were men, 33% were women, and 12% were children and young adolescents.

### Global progress made in TB prevention and treatment

Ending the global TB epidemic remains a distant goal, but several positive trends following strong post-COVID recovery efforts continued in 2023.

1. The global rise in the number of people falling ill with TB each year has slowed and started to stabilize.
2. The global number of people dying from TB each year continues to fall.
3. The WHO African and European regions have made good progress toward the 2025 milestones for reductions in the TB incidence rate and the number of deaths caused by TB.
4. The globally reported number of people newly diagnosed with TB reached a new high in 2023.
5. The treatment success rate for people with drug-susceptible TB has been sustained at a high level and continues to improve for people with drug-resistant TB.
6. The coverage of TB preventive treatment has been sustained for people living with HIV and continues to improve for household contacts of people diagnosed with TB (Table 1).

**Table 1:** Summary of progress toward new global targets for 2027.

	Progress towards new global targets for 2027
1	Coverage of rapid testing for TB: target 100% of those newly diagnosed; status in 2023, 48%
2	TB treatment coverage: target 90%; status in 2023, 75%
3	Coverage of TB preventive treatment: target 90% among high-risk populations; status in 2023, 21% among household contacts of people diagnosed with TB, 56% among people living with HIV
4	Availability of a new TB vaccine that is safe and effective: target, preferably within five years; status in 2023, six vaccines in Phase III trials
5	Funding for TB prevention, diagnostic and treatment services: target US$ 22 billion; status in 2023, US$ 5.7 billion
6	Funding for TB research: target US$ 5 billion; status in 2022, US$ 1.0 billion

Source: Global TB Report 2024 (2).

## Discussion

### The role of community networks in TB case detection

Early detection of TB cases is crucial to controlling the spread of the disease and improving patient outcomes. In many communities, particularly in rural and underserved areas, access to formal healthcare services can be limited, leading to delays in diagnosis and treatment. Community networks, however, offer a powerful tool for enhancing case detection by leveraging local knowledge and social ties (9, 10, 13).

Studies have shown that CHWs play a pivotal role in detecting TB cases within their communities. By conducting door-to-door visits, organizing community screening events, and educating the public about TB symptoms, CHWs can identify potential cases earlier and refer individuals to healthcare facilities for diagnosis (14). For example, a study conducted in Uganda found that CHWs were able to identify more TB cases in rural communities than traditional health services, highlighting the importance of localized, community-based interventions (15). Furthermore, training and empowerment is needed for CHWs to be able to recognize TB symptoms and refer individuals for testing and to conduct community-based screening (utilizing mobile clinics, outreach teams, and door-to-door screening) in endemic areas, as well as conducting targeted screening in high-risk populations (e.g., contacts of TB patients, people living with HIV, miners, and prisoners), has been proven to be effective for early TB detection (16).

In addition to CHWs, other community stakeholders, such as local leaders and volunteers, contribute to TB detection efforts. These individuals, often well-respected and trusted by their communities, can encourage individuals to seek care when symptoms arise and help disseminate accurate information about TB. Furthermore, community-based organizations (CBOs) and NGOs often collaborate with healthcare providers to create networks that increase awareness and facilitate access to diagnostic services (17).

Despite the effectiveness of community networks in TB case detection, several challenges remain. The stigma associated with TB can prevent individuals from coming forward for testing, particularly in communities where TB is highly stigmatized due to its association with poverty, HIV/AIDS, or fear of isolation. The stigma also creates a barrier for the community worker who is working for the welfare of TB patients (11, 18). Overcoming stigma requires concerted efforts to educate communities about the nature of TB, its treatment, and the importance of early detection (19). Culturally sensitive interventions that involve local stakeholders can help address these challenges and create an environment where individuals feel safe seeking care.

### Supporting treatment adherence through community engagement

TB treatment regimens are lengthy, often requiring patients to take medication for 6 months or more. Treatment adherence is essential to prevent drug resistance, relapse, and further transmission. However, maintaining adherence is challenging, particularly for patients who face socio-economic barriers, experience side effects from medication, or live far from healthcare facilities.

Community engagement has proven to be a valuable strategy in supporting TB patients throughout their treatment journey. CHWs and community volunteers often provide directly observed treatment (DOT) services, monitoring patients taking their medication and offering support and encouragement. This personalized approach has improved treatment adherence, particularly in settings where patients may struggle to complete their regimen (20).

Moreover, community-based support groups provide a platform for TB patients to share their experiences, challenges, and successes with others facing similar circumstances. These support groups can help reduce feelings of isolation and empower patients to stay committed to their treatment. In a study conducted in Ethiopia, patients who participated in community-based TB clubs reported higher levels of adherence and improved mental health outcomes compared to those who did not participate in such groups (21).

Financial and social support also play a critical role in treatment adherence. Community networks (such as TB youth networks, women’s groups, and farmers’ groups) can facilitate access to resources such as transportation to healthcare facilities, food assistance, and financial aid for patients who cannot work during their treatment. Partnerships with local businesses, NGOs, and government agencies can help mobilize these resources, ensuring TB patients receive the support they need to complete their treatment (22). India has pioneered innovative community-based TB management models, notably the Nikshay Poshan Yojana and Nikshay Mitra initiatives (23, 24). These programs align with global strategies and offer best practices adaptable to other settings. The Nikshay Poshan Yojana (NPY)—Nutritional Support for TB Patients concept launched in 2018 (by the Government of India under the National TB Elimination Program—NTEP) to provide direct financial support to TB patients to improve treatment adherence and recovery has proven to improve treatment adherence by reducing economic barriers, reducing malnutrition-related complications that hinder TB recovery, and empowering marginalized communities, ensuring better access to care in India (23). In addition, the Nikshay Mitra—Community Engagement in TB Management, launched in 2022 (as part of India’s TB Elimination Mission) to encourage individuals, corporations, and civil society to “adopt” and support TB patients with nutritional and psychosocial assistance, has mobilized over 75,000 Nikshay Mitras, covering hundreds of thousands of TB patients, strengthened community ownership in TB care, and encouraged corporate social responsibility (CSR) involvement (24).

However, the success of community engagement in supporting treatment adherence is often hampered by resource limitations. In many low-income settings, CHWs are overburdened, underpaid, or lack the necessary training and resources to support TB patients effectively. Strengthening the capacity of CHWs and ensuring they have access to adequate resources is essential for sustaining these community-based efforts (25).

### Reducing TB-related stigma through community interventions

Stigma remains a significant barrier to effective TB case management, as it can prevent individuals from seeking care, adhering to treatment, or engaging with community support networks. TB-related stigma is often intertwined with social and economic factors, such as poverty, HIV status, and cultural beliefs about illness and contagion (26). Addressing this stigma is essential to improving TB outcomes and requires targeted, community-based interventions.

Community engagement plays a crucial role in reducing TB-related stigma. Public education campaigns, led by local leaders, CHWs, and NGOs, can challenge misconceptions about TB and promote understanding of the disease as a curable and manageable condition. These campaigns often use a variety of platforms, including radio, social media, and community events, to reach a broad audience and encourage positive attitudes toward TB patients (27–29).

In some cases, former TB patients who have completed treatment become advocates and peer educators within their communities. By sharing their experiences and emphasizing the importance of seeking care, these individuals help reduce the fear and shame often associated with TB. In South Africa, TB survivors have been trained as “treatment ambassadors” to support newly diagnosed patients and raise awareness about the disease in their communities (11, 18, 26).

Despite these efforts, stigma reduction remains challenging, particularly in regions where TB is highly prevalent and deeply stigmatized. Structural factors, such as social inequality, limited access to healthcare, and cultural norms, contribute to the persistence of stigma. Addressing these broader determinants of health is critical to creating a more supportive environment for TB patients (5).

### Multi-sectoral collaboration and sustainability

Sustainable TB case management requires collaboration across multiple sectors, including healthcare institutions, government agencies, civil society organizations, and the private sector. Building strong community networks involves fostering partnerships between these stakeholders to ensure that TB programs are well-resourced, culturally appropriate, and responsive to local needs.

Multi-sectoral collaboration is particularly important in addressing the social determinants of TB, such as poverty, housing, and malnutrition, which contribute to the spread of the disease and affect treatment outcomes (20, 30). Governments and NGOs are critical in creating policies that address these factors, while healthcare providers focus on delivering clinical care. In many successful TB programs, local businesses, schools, and religious organizations are also engaged to provide support and resources for TB patients and their families (31).

However, sustaining these collaborations over time can be challenging. Limited funding, competing priorities, and weak governance structures can hinder the development of long-term, effective partnerships. Ensuring the sustainability of community networks for TB case management requires ongoing investment in capacity building, financial resources, and policy frameworks prioritizing TB control (12).

## Conclusion

Urgent action is required to end the global TB epidemic by 2030, a goal adopted by all member states of the United Nations (UN) and the World Health Organization (WHO). Community networks and engagement play a vital role in effective TB case management by supporting early case detection, improving treatment adherence, reducing stigma, and fostering collaboration across sectors. India has pioneered innovative community-based TB management models, notably the Nikshay Poshan Yojana and Nikshay Mitra initiatives. These programs align with global strategies and offer best practices adaptable to other settings.

The involvement of CHWs, local leaders, NGOs, and community-based organizations enhances the reach and effectiveness of TB programs, particularly in resource-limited settings. Despite the significant contributions of community networks, challenges remain in sustaining these efforts. Stigma, resource limitations, and structural inequalities continue to impede progress in TB control. Addressing these challenges requires a comprehensive approach that includes investment in community health infrastructure, multi-sectoral collaboration, and culturally tailored interventions.

Future strategies for TB control should prioritize strengthening community networks, ensuring that these networks are well-resourced, adequately trained, and empowered to contribute to the fight against TB. By leveraging the power of community engagement, healthcare systems can improve TB outcomes and move closer to achieving global TB elimination goals.

## Data Availability

The original contributions presented in the study are included in the article/supplementary material, further inquiries can be directed to the corresponding author.
